# Ingested Potassium Chloride Pills on Imaging Misdiagnosed As Foreign Bodies in the Stomach: An Insight on Radiopaque/Hyperdense Substances in the Gastrointestinal Tract

**DOI:** 10.7759/cureus.27116

**Published:** 2022-07-21

**Authors:** Samyak Dhruv, Kuldeepsinh P Atodaria, Woo Jin Seog, Ahmed A Hassan

**Affiliations:** 1 Internal Medicine, MedStar Health, Washington DC, USA; 2 Internal Medicine, Jefferson Abington Hospital, Abington, USA; 3 Internal Medicine, Touro College of Osteopathic Medicine, New York, USA; 4 Internal Medicine, Staten Island University Hospital, New York, USA

**Keywords:** general radiology, radioopaque, upper endoscopy, foreign bodies, potassium chloride

## Abstract

Foreign bodies are very common in the GI tract. Around 100,000 cases are reported each year in the United States. A total of 80% of those foreign body ingestions occur in the pediatric population. There are several reasons for foreign body impaction in the GI tract in adults. Psychiatric problems, anatomical abnormalities in the GI tract such as esophageal web, diverticula, stricture, and eating big food boluses are frequent causes of foreign body impaction in adults. Rarely do radio-opaque ingested materials appear as a foreign body in imaging studies. Such objects include several commonly used medications such as iron preparations, potassium chloride pills, amiodarone, spironolactone, bisoprolol, and lisinopril. Herein, we present one such case of potassium chloride pill ingestion, where it appeared as a foreign body in the stomach. However, on the endoscopic examination and repeat X-ray, the foreign body had been digested and disappeared.

## Introduction

Around 100,000 cases of GI foreign bodies are reported each year in the United States. Eighty percent of those cases are seen in the pediatric population. Foreign body ingestion is, in fact, one of the most common reasons for pediatric ER visits. Instances of adult patients who present for foreign body ingestion are usually those with psychiatric disorders, substance dependence, intellectual disability, or from prison settings. Causes for impacted ingested materials are a patient with a history of anatomical abnormalities, such as esophageal web, diverticula, stricture, eosinophilic esophagitis, neurological disorders and associated dysphagia, and consumption of large food boluses. Around 80-90% of foreign bodies will pass spontaneously, while 10-20% of instances require endoscopic intervention, and about 1% require surgical retrieval [1].

Foreign body ingestion is often clinically diagnosed while supplemented and confirmed with imaging studies. X-ray is the first choice in imaging. A tomographic study or endoscopy is done if there are concerns of complications from ingestion or an inability to reach a definitive diagnosis. The management is adjusted depending on the nature of ingested material, location within the GI tract, and time since ingestion [2]. However, it is essential to note that there are instances of certain oral medications that can also appear radio-opaque on imaging and will not require any intervention. In this case, ingested potassium tablets incidentally discovered on imaging were initially thought to be metallic foreign bodies.

## Case presentation

**Figure 1:**
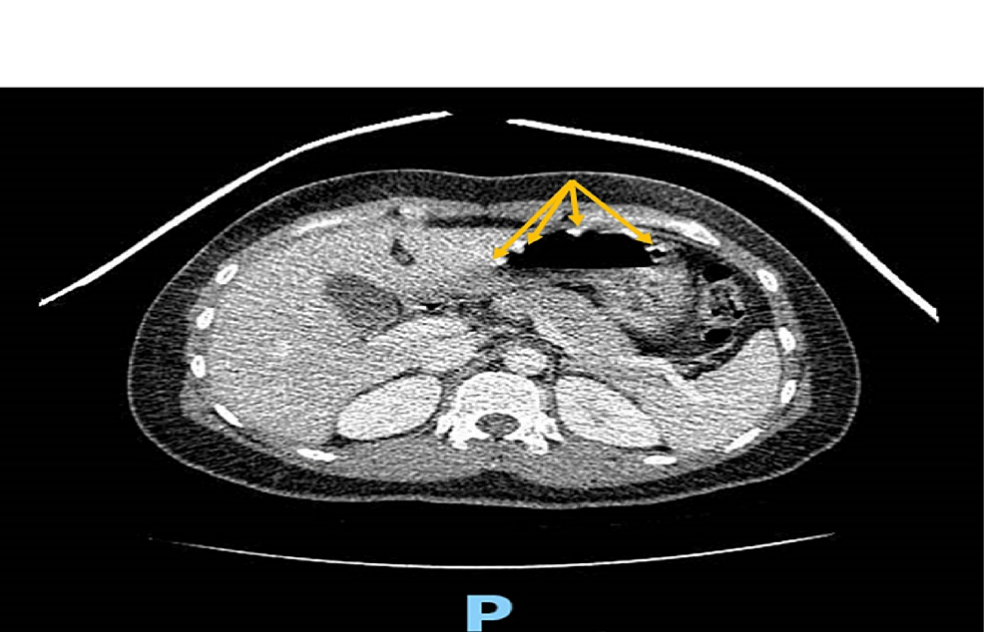
CT scan of the abdomen showing four hyperdense substances (yellow arrows) in the stomach.

**Figure 2:**
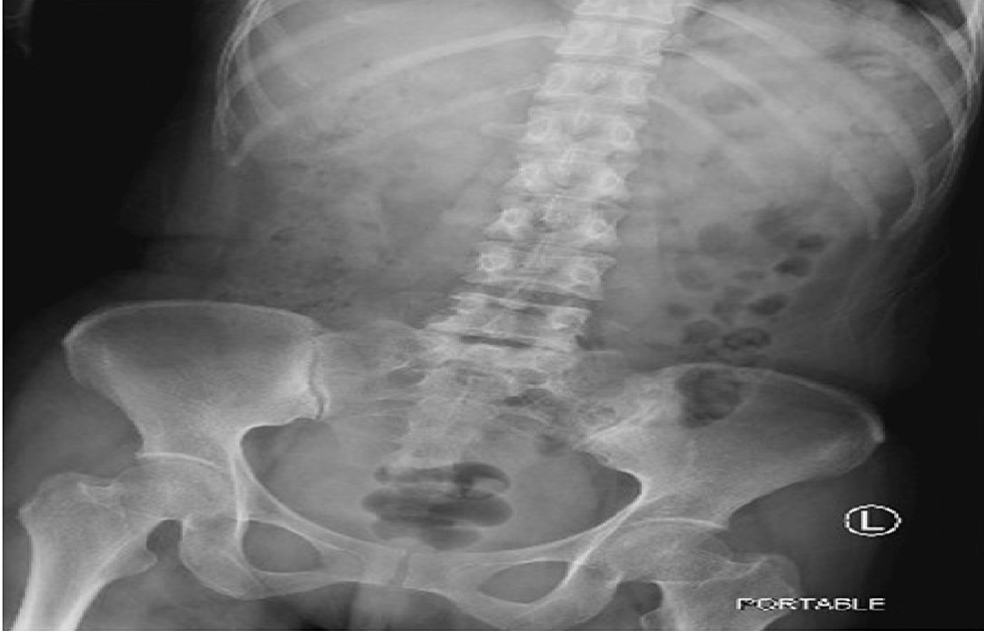
X-ray abdomen fails to identify any radiopaque substances.

A 36-year-old woman with no known past medical history presented to the ED with a complaint of epigastric abdominal pain associated with burning chest pain of three weeks' duration. She stated that her pain was worse in the mornings. The pain was worsened by the intake of spicy food and not relieved by famotidine. A CT scan of the abdomen and pelvis was obtained to assess the intraabdominal organs further and showed findings described as tiny four metallic irregular objects in the antrum of the stomach consistent with ingested materials or foreign bodies (Figure 1). An esophagogastroduodenoscopy (EGD) was performed the following day. It showed two black pigmented antral ulcers (1 cm and 0.7 cm in diameter) with no active bleeding and mucosal erythema compatible with non-erosive gastritis. The foreign bodies described on the CT scan from the previous day were no longer seen in the stomach during the EGD. An abdominal X-ray performed the next day did not demonstrate any metallic or radio-opaque objects in the stomach (Figure 2). Therefore, no overt passage of any foreign body occurred during the patient's hospital stay. Upon further investigation of the patient's hospital stay, it was discovered that the patient was given potassium chloride tablets in the ER for low potassium levels shortly before going for the CT scan. Therefore, it was determined that the objects described on the CT scan were likely the ingested potassium chloride pills which had later dissolved before the EGD. The patient was discharged from the hospital with a diagnosis of peptic ulcer disease and followed up with GI outpatient. The results of biopsies obtained during the EGD showed gastritis and confirmed the presence of Helicobacter pylori. The patient was started on quadruple therapy and reported improvement in her symptoms on subsequent follow-up visits.

## Discussion

Foreign body ingestion is a common cause of ED visits. It is more common in the pediatric population but can also be seen in adults. In the adult population, those who have no teeth, have psychiatric disorders, have intellectual disorders, or are under the influence of substances and use it as a form of smuggling contraband are most likely to present to the ED for foreign body ingestion. Some commonly identified ingested foreign bodies include impacted food boluses, fishbones, metallic objects such as pins and coins, dental fillings, toothpicks, and batteries [3].

While 90% of ingested foreign bodies that enter the stomach pass without intervention, some require endoscopic or surgical removal [4]. Less than 1% of foreign bodies that enter the stomach can cause intestinal perforation, with the exception of sharp or pointed objects having an intestinal perforation rate of up to 35% [5]. Some complications from foreign body ingestion can include lacerations, ulcers, perforation, obstruction, and fistula formation. The type and size of the object and the duration of impaction are independent risk factors for the development of complications [6].

Although ingested foreign bodies that have entered the stomach will pass through the GI tract often without complications, it is crucial to identify and characterize the foreign body as it can affect management. For example, batteries remaining in the stomach for more than 48 hours require endoscopic removal as it can cause mucosal burns with ulcers and bleeding, while ingested smuggled narcotic drug packets are not advised to be removed due to concerns of rupture [5]. Sharp-pointed objects in the stomach or duodenum should be removed due to concerns of perforation. Objects that are larger than 2.5 centimeters should also be removed due to potentially being unable to pass the pylorus, and objects greater than 6 centimeters in length at or above the proximal duodenum may need to be removed due to difficulty passing through the curvature of the duodenum. Hence, it is best to identify and characterize the foreign object to predict possible complications or initiate the need for intervention.

Initial evaluation often involves plain radiography, which can identify the presence, nature, number, and location of some foreign objects [7]. However, a plain radiograph without abnormal findings does not exclude the presence of foreign bodies. CT can provide more information about the foreign body and better evaluate for the presence of complications, but it may not be able to detect radiolucent objects [2]. CT is generally utilized when perforation is suspected and with the ingestion of sharp objects. Some hyperdense foreign objects that can be found on CT scans include dentures, most animal bones, bezoars, heavy metals and glass, gallstones, dislodged feeding tubes, and endoscopic capsules [8,9]. Certain medication tablets can appear radio-opaque in imaging studies, mimicking a foreign body. Sieron DA et al. have recently measured the CT density of the 50 most commonly used medications by CT spectroscopy, and the top five most radio-dense pills were as follows: 1) Cordarone (Amiodarone), 2) Potassium chloride pills, 3) Concor (Bisoprolol), 4) Aldactone (Spironolactone), 5) Lisinopril [10]. Other common medications that have been mistaken as foreign bodies are multivitamins, iron supplements, and sustained-release medications. The radiodensity of the pills on imaging can also vary depending on the enteric coating of the pills, the orientation of the pills, the presence of adjacent air contrasting the pills, and the size of the patient [11]. The timeframe from ingestion should also be considered as many of these medications can remain radio-opaque for up to six hours [12].

Potassium preparations are among the most consistently radio-opaque ingested medications [11]. This can sometimes result in incidental imaging findings that can be mischaracterized as metallic objects or even mimic active bleeding. In one case, degrading potassium chloride pills ingested five hours before a CT angiography of the abdomen were mistaken for an active gastric bleed due to its high radiodensity, mimicking contrast extravasation [13]. Therefore, gastroenterologists and interventional radiologists should be aware of this entity to avoid unnecessary intervention.

In conclusion, it is necessary to consider any recent medication ingestion when interpreting scans with radio-dense foreign bodies. The lack of this consideration when interpreting scans can lead to misdiagnosis and affect upcoming treatment. There have been instances where radio-dense medications have been misinterpreted as GI fistulas, urinary tract stones, or pancreatic calcifications [14-16]. There have also been instances where the presence of such pills distracts from other abnormalities responsible for the true reason for a patient's complaints [17]. In our case, the patient had an initial presentation of abdominal pain with imaging showing radio-dense objects, prompting the suspicion that such objects were the cause of the patient's complaint. However, during the endoscopy, the objects were not seen; instead, the gastric ulcers were identified. The objects were likely potassium chloride pills that had later dissolved, while the cause of the patient's abdominal pain was peptic ulcer disease.

## Conclusions

Foreign body ingestion is a common complaint for patients presenting to the ER, and characterization of the foreign body from imaging can affect the patient's management. In our case, consideration of the temporal relationship between the administration of oral potassium repletion and imaging performed after that was needed to avoid misinterpretations and improper diagnoses from imaging. In the adult population, these misinterpretations become especially significant in patients with a history of psychiatric illnesses or those with a higher likelihood of presenting for foreign body ingestion and can be avoided by increasing our suspicion and changing management. Therefore, we recommend that it should be a general practice to rule out any therapeutic medications as a cause of radio-dense substances identified in the imaging studies before labeling it as foreign body ingestion or active GI bleed.
